# Newly Standing Infants Increase Postural Stability When Performing a Supra-Postural Task

**DOI:** 10.1371/journal.pone.0071288

**Published:** 2013-08-05

**Authors:** Laura J. Claxton, Jeffrey M. Haddad, Katelyn Ponto, Joong Hyun Ryu, Sean C. Newcomer

**Affiliations:** 1 Department of Health and Kinesiology, Purdue University, West Lafayette, Indiana, United States of America; 2 Center for Aging and the Life Course, Purdue University, West Lafayette, Indiana, United States of America; 3 Department of Kinesiology, California State University San Marcos, San Marcos, California, United States of America; University of California, Merced, United States of America

## Abstract

Independent stance is one of the most difficult motor milestones to achieve. Newly standing infants exhibit exaggerated body movements and can only stand for a brief amount of time. Given the difficult nature of bipedal stance, these unstable characteristics are slow to improve. However, we demonstrate that infants can increase their stability when engaged in a standing goal-directed task. Infants' balance was measured while standing and while standing and holding a visually attractive toy. When holding the toy, infants stood for a longer period of time, exhibited less body sway, and more mature postural dynamics. These results demonstrate that even with limited standing experience, infants can stabilize posture to facilitate performance of a concurrent task.

## Introduction

Humans are the only extant primate to locomote exclusively with two legs, making bipedalism a main characteristic defining humanity. Although the exact evolutionary forces that led to the development of bipedalism are debatable, most theories suggest that when our non-human ancestors first stood upright they gained many advantages over other animals. One advantage of bipedalism is that locomotion is possible without the need to use the upper limbs [1]–[3]. Once the hands were relieved from the burden of balance and locomotion, they could be used to wield tools, simplifying many daily tasks necessary for survival (e.g., cultivation, defense). Although the mastery of tool-use allowed humans to become the most adaptable and dominant species to ever inhabit the planet, it came at a cost. Specifically, human infants are extremely altricial [4], [5] and in most cases cannot stand on their own until around 10- to 11-months of age.

### The Development of Upright Stance

The ability to stand is a critical component of development that is necessary before infants can perform goal-directed behaviors such as reaching and walking [6]. Despite the importance of posture to the overall motor development of the child, minimal research has investigated posture in newly standing infants. Rather, the majority of postural development studies have examined infants who could stand for a long period of time. These studies typically require infants to stand on a force plate as various sensory manipulations (e.g. visual or tactile) are performed [7], [8]. Center of pressure (CoP) – the instantaneous location of the vertical ground reaction force vector – is then assessed using a variety of spatial and temporal measures to make inferences regarding postural stability and the ability of infants to integrate and utilize sensory information to maintain stance. Interestingly, direct measures of postural stability (i.e. how long an infant can remain standing) are typically not assessed. In general, analysis of the CoP time series has revealed that both the control and dynamics of infant postural sway is very immature compared to adults and older children. Specifically, young infants exhibit a greater magnitude of postural sway that has a higher frequency and velocity compared to infants with experience remaining upright. Although posture improves with standing experience, e.g. [9], the immature postural dynamics discussed above appear to persist through childhood [10]–[12].

Because posture is unstable early after the emergence of upright stance, the ability to efficiently locomote is still months away. For balance to improve, infants have the difficult task of learning to control and coordinate their multiple degrees of freedom (i.e. muscles, joints, and motor units) so that a relatively high center of mass remains within a small base of support. To further complicate matters, infant's body proportions are rapidly changing. Thus, control strategies that work at one point in time may not be appropriate a short time later. Most engineers and roboticists are well aware of the inherent difficulties associated with bipedalism and despite advances in technology have yet to develop a mechanical biped with the mobility and adaptability enjoyed by most humans.

Given the inherent difficulties of adopting a bipedal posture, a major emphasis of previous research has been to investigate the underlying mechanisms that lead to the development of mature postural control. Mature postural control requires the effective utilization of various prospective and reactive strategies to maintain balance [13]. Prospective strategies involve adjusting posture to attenuate potential predicted threats to balance. For example, adults will activate posterior postural muscles in the trunk and leg prior to initiating a rapid arm raising movement [14]. This allows the body to become stabilized prior to an internally generated perturbation. These prospective strategies begin to emerge in infancy and are refined with development [15]. Reactive control strategies occur after the body has been perturbed in some way. A number of feedback driven reactive strategies exist, including reflexive, preprogrammed, or voluntary responses, e.g. [16]. Given that reactive strategies are feedback driven, proper function requires sensory information (detection of the balance threat) to be integrated with the proper motor response within a reasonable time delay [17]–[19]. The sensory integration needed for proper feedback postural control begins to develop early in infancy [20]. Sitting infants modify postural movements based on visual information such as a moving room [21], [22]. Additionally, infants reduce sway when provided with light touch feedback, indicating proprioceptive feedback is being used to stabilize posture [23].

### The Development of Task-Dependent Postural Control

Developing the ability to control posture in a manner that supports the performance of other goal-directed tasks is important since most daily activities are performed while standing [24], [25]. For example, adults minimize body sway when performing a manual task that requires a high degree of precision compared to a task requiring little precision since any extraneous body sway could result in the hand making less accurate movements [26]. Learning to control posture based on the context of a concurrent task is likely an important aspect of motor development [6], [27].

Limited research has examined if and when infants stabilize posture and stand for a longer duration of time when given a task to perform. In a recent study, it was found using Stabilogram Diffusion Analysis that with limited standing experience, there is an increased influence of short time-scale components to maintain balance [9]. The authors suggest that these short time-scale components indicate that infants have not yet developed a sufficient internal model of standing. Without an appropriately formed internal model, it would be expected that newly standing infants would simply react to internally and externally generated perturbations rather that adaptively control posture to effectively complete a goal-directed task.

Although newly standing infants may lack a sufficient internal postural model, we recently found infants are capable of modulating the dynamics of postural sway based on the demands of a task [27]. Specifically, when holding a toy, infants exhibit more complex sway patterns, indicating they are recruiting more functional degrees of freedom when standing and performing a goal-directed task. This increase utilization of the body's degrees of freedom my allow infants to more effectively interact with the toy. However, when not holding a toy, infants appear to exhibit a more exploratory postural strategy. Specifically, they employ a strategy whereby their base of support was systematically probed and explored. This strategy may be a mechanism utilized by infants to learn about the dynamical interaction between their body and the environment [27]. The changes in postural sway when interacting with a toy suggest that the balance abilities of newly standing infants are better than they appear and that newly standing infants can indeed stabilize posture to perform a goal-directed task.

In general, previous literature examining task-dependent postural control has not directly assessed postural stability. Rather, center of pressure movements are examined to make inferences regarding postural stability, e.g. [24], [25], [27]. One issue with making interpretations regarding stability using the CoP time series is that the infant often never becomes unstable or falls during data collection. Although developmental research has typically considered an increase in postural sway to relate to less postural stability, for many reasons this interpretation can be ambiguous. First, from a mechanical perspective, stability is a binary state. An individual or object is either stable or unable to remain upright. Assessing properties of the time series does not directly provide information regarding this binary state. For example, an individual can exhibit large magnitudes of sway, yet never fall. Second, in the adult literature, an increase in postural sway can sometimes reflect a more flexible postural system (i.e. healthy and more stable) [28]. Finally, as mentioned above, increased postural movements can be indicative of exploratory postural strategies. Thus, although infants may exhibit apparently immature postural movements, these movements are helping them learn how to control their bodies within a dynamically changing environment [27]. Given that improvements in the movement repertoire of infants is occurring concurrent with physical growth and maturation, exploratory postural movements that help infants learn motor skills may be particularly beneficial.

Karasik, Adolph, Tamis-LeMonda, and Zuckerman [29] is one of the only studies to directly assess stability of crawling and walking infants as they performed a goal-directed task. In their study, infants were observed while either carrying or not carrying a toy. They found infants fell twice as much when walking without carrying an object. Thus, it appears walking infants do modulate postural stability based on the demands of a goal-directed manual task. This increased stability may improve the ability of the infant to attend to the toy. These findings were interesting since the infants studied had limited locomotory experience. It is unknown if infants' ability to stabilize posture to facilitate the performance of a goal-directed task emerges at earlier milestones (i.e., independent standing) or if this rather sophisticated behavior only emerges after months of standing experience. The purpose of this study was therefore to examine if newly standing infants, who typically stand in an unstable posture, would become more stable (stand for a longer period of time) when given a task requiring stability. Based on our previous study [27] which demonstrated changes in the structure of postural sway when interacting with a toy, we hypothesized infants would stand longer when holding a toy. This would suggest the ability to modulate posture based on the demands of a concurrent task emerges soon after the acquisition of independent stance.

## Methods

The Purdue University Institutional Review Board approved all procedures and parents signed an informed consent form prior to participation.

### Participants

Sixteen infants (9 females; *M* age = 11 mo, 3 wks; *range* = 9;2 to 13;1) were recruited to participate. All infants were capable of standing unsupported for 2–3 seconds and were unable to take more than a couple of independent steps. Thus, all infants were at a similar stage of motor development. Potential participants were first identified from birth announcements published in the local newspaper. A recruitment letter detailing the research was then sent to parents. Parents received an infant t-shirt as a token of appreciation. The data used in this study was part of a larger data set. Thus, the subjects and data collection were the same as those used in [27]. However, the data analyses, dependent variables, and interpretations were different in each study.

### Procedure

Infants performed two conditions while standing on a force plate (AMTI; Watertown, MA). The force plate recorded the reaction forces and moments exerted on the ground by the infant. Force plate data were used to calculate center of pressure (CoP) and the amount of time the infant was able to independently stand. Force plate data were recorded at 120 Hz and filtered using an 8 Hz low pass Butterworth filter. The CoP is the point location of the vertical ground reaction force vector and is used to determine the magnitude of body sway. The CoP time series was used to calculate various variables (described below in the assessments and measures section). The force plate data were also time synchronized with a digital video camera.

Two conditions were performed: 1) no toy and 2) toy-hold. In the no toy condition, the infant simply stood on the force plate. In the toy-hold condition, the experimenter handed the infant a toy once they were standing on the force plate. In both conditions, the experimenter or parent either lowered the infant onto the force plate in a standing position or allowed the infants to pull themselves into a standing position using a chair that was placed next to the force plate. In both conditions, parents remained close to the infant and encouraged them to stand. They were also instructed to provide the infant with support once they began to lose balance. Four trials in each condition were performed in alternating order. Only trials where the infant was compliant (stood on the force plate) were further analyzed. All infants in the final sample performed at least 3 trials of each condition. Data from all trials in each condition were used in the final sample. A different age-appropriate toy - rattle (60 g), duck (60 g), toy phone (90 g), and toy keys (70 g) - was used in each toy-hold trial so the infant would not become bored with a particular toy. The weights of the toys were assessed using a pediatric strain gauge scale. The graspable area of all toys ranged from 1.2–1.4 cm in circumference. The typical inside hand circumference (50th percentile) for one-year-olds is 2.1 cm [30]. Thus, the toys were easily graspable.

### Assessments and Measures

The force and moment data collected from the force plate were used to calculate the duration of independent stance and the center of pressure (CoP). The CoP time series was then used to calculate various time-dependent and time-independent measures of postural sway. Time-independent measures are typically spatial in nature and therefore not sensitive to the temporal evolution of the CoP signal. Time-dependent measures however are influenced by the temporal evolution of the signal. For example, if we compare a 1 Hz sine wave (amplitude = 1V, and no bias) to a shuffled version of the same sine wave, the two signals would obviously look very different. Specifically, the shuffled sine wave would visually appear to be random noise. Despite the visual differences, the two signals would have the same range (2V), standard deviation (.707) and average (0). This is because these measures are time-independent. In order to assess the structural differences between the signals, it is necessary to compute time-dependent measures. The details of how each measure was calculated are described below.

#### Duration of independent stance

The duration of independent stance was defined as the time period between stance onset and when the infant lost balance. In the no-toy condition, independent stance onset was identified as the point in time when the infant was standing and completely supporting their own body weight (the magnitude of the vertical ground reaction force vector matched the infant's body weight). In the toy-hold condition, independent stance onset was identified as the instant in time when the infant grasped the toy from the experimenter (identified from a synchronized digital video recording) and when the magnitude of the vertical ground reaction force vector matched the infant's body weight. In both conditions, independent stance offset was identified as the instant in time when the magnitude of the vertical ground reaction force vector began to decrease (indicating the infant's center of mass was accelerating towards the ground). The force plate data is capable of detecting changes in Fz at a high spatial and temporal resolution. Thus, we were able to detect the end of the trial if infants exhibited either a controlled (lowering themselves to a sitting position) or uncontrolled fall. The times of independent stance onset and offset were confirmed using the synchronized digital video recording. A reliability coder examined 50% of the onset and offset times. There was an 85% agreement for trial onset times (within 250 msec) and an 89% agreement for trial offset times (within 250 msec). A third coder resolved any disagreements.

#### Time-dependent and time-independent measures of postural sway

The time-dependent and time-independent CoP measures were calculated over the duration of time the infants remained standing upright. Thus, the epochs of time where children exhibited excessive arm, head, hip or knee movements, as identified from examining the Fz time series and synchronized digital video. For example hip or knee flexion resulted in deviations of the Fz time series. Head and arm movements were identified from the digital video. All statistical analyses were performed using paired samples *t*-tests.

In the current study, the time-dependent measures calculated were Recurrence Quantification Analysis (RQA) and the average velocity of sway. To determine CoP velocity, a net CoP distance time series was calculated by determining the Euclidian distance the CoP trajectory traveled between successive data points. The net CoP distance time series was then differentiated using a first order central difference technique to yield CoP velocity. The velocity values were then averaged across the entire time the infant was standing to calculate the average velocity for each trial. Sway velocity has been used in previous infant standing research and has been shown to improve with development, e.g. [8]. Additionally, average velocity of the CoP provides a time-normalized measure of the degree to which the infant's body moved. The second time-dependent analysis performed was RQA (a measure that examines the higher-order structure of the CoP time series). RQA was assessed in both the anterior-posterior and medial-lateral directions. RQA has previously been used to reveal properties of the CoP signal such as the degree of randomness (called percent determinism), entropy, and dynamic stability [31]–[33]. It is important to note that many other non-linear measures have been used in past research to analyze the CoP signal. These measures have provided valuable insight into the development, adaptability, and stability of posture [34]. However, many of these measures are sensitive to the length of the time series. Thus, in the present study, these measures were not appropriate since newly standing infants only remain upright for a short amount of time. Fortunately, RQA is less sensitive to the length of the data series.

**Figure 1:**
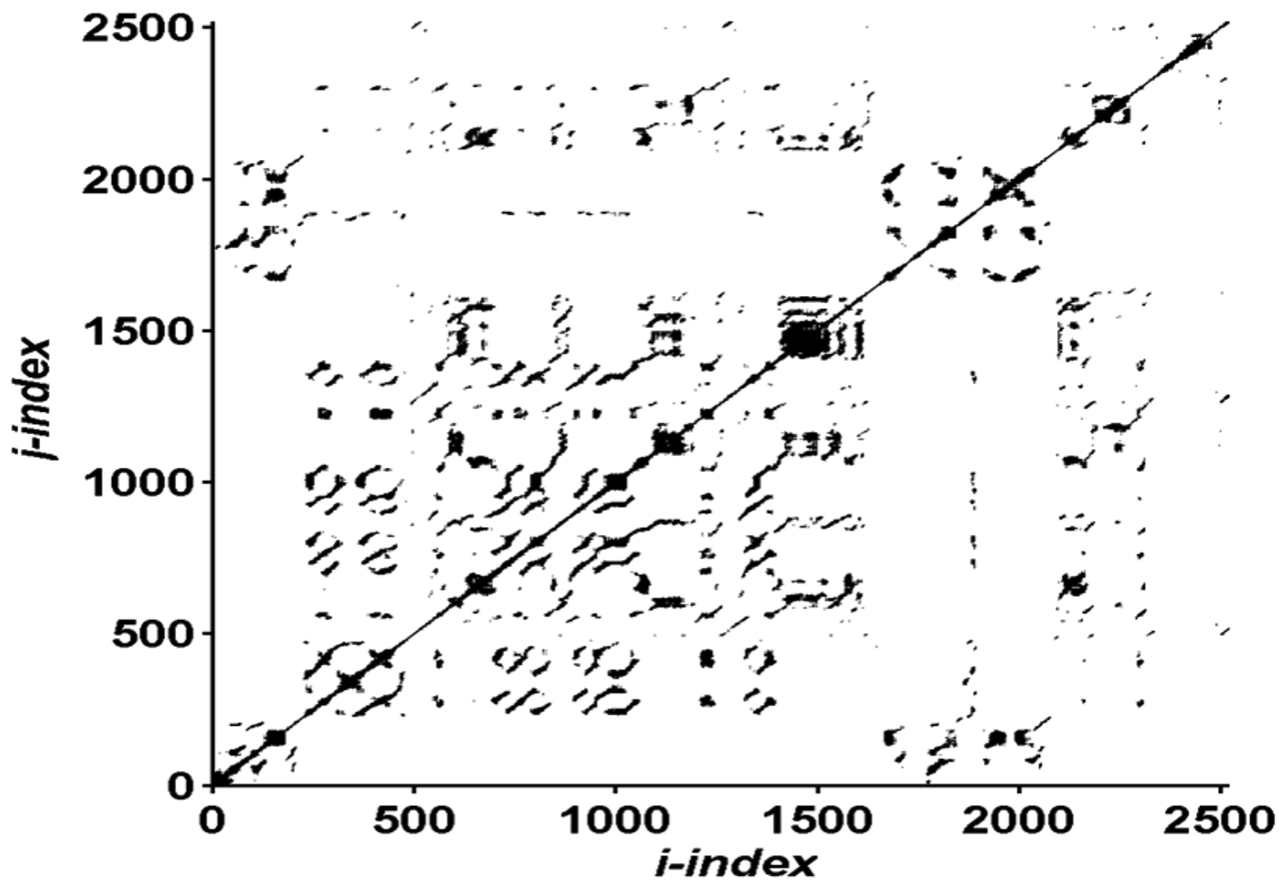
Example recurrence plot from the CoP time series in the medial-lateral direction in the toy-hold condition. The dark dots represent the points that were recurrent. In this plot the percentage of recurrence points was 2.5%. The percent determinism, the main RQA variable reported here, was 79.6%. The percent determinism was calculated as the percent of recurrence points that were in diagonal line structures.

In order to perform RQA analysis, the original time series was first reconstructed into a 4 dimensional state space using a time lag of 5 points. The embedding dimension of the reconstructed state space and the time lag were assessed using the mutual information and false nearest neighbors technique respectively. A matrix of the Euclidian distance between all possible points in the multi-dimensional state space (normalized to the mean distance) was then constructed. Finally, a recurrence plot was constructed from the distance matrix. The recurrence plot identifies points that are similar (recurrent) within a given threshold (see Figure 1). In the current study, the radius threshold used was 10 data points. The radius threshold of 10 was used because it resulted in the total percent or recurrence points remaining under 5%, a threshold commonly used in CoP data, e.g. [33]. The radius threshold is essentially a slop value that accounts for the fact that two points are never exactly the same. Multiple outcome variables can be calculated by quantifying the structure of the recurrence matrix. In the current study we quantified the percent determinism. Percent determinism provides insights regarding the predictability of the signal and is calculated as the proportion of points in the recurrence matrix that form diagonal line structures. A line structure was defined using a minimum threshold of two data points. In previous postural research additional outcome measures from the RQA plot, including Linemax, entropy, and percent recurrence have been calculated. We chose not to assess these variables for various conceptual and methodological reasons. Specifically, Linemax, the RQA variable used to measure dynamic stability, was not used because it is not appropriate on data sets that have trials of varying length. This is because Linemax is calculated as the longest diagonal line segment in the recurrence plot and is not scaled to a relative value of the data length. The percent of recurrence points was not used because this variable is directly manipulated by changing the radius threshold. Finally, RQA entropy was not used given it is difficult to interpret because it relates to the complexity of the recurrence plot rather than the time series. Specifically, RQA entropy assesses the distribution of line structures of varying length. The standard deviation of the CoP signal (CoPSD) in both the anterior-posterior and medial-lateral directions was assessed as a time independent measure of posture.

## Results

**Figure 2:**
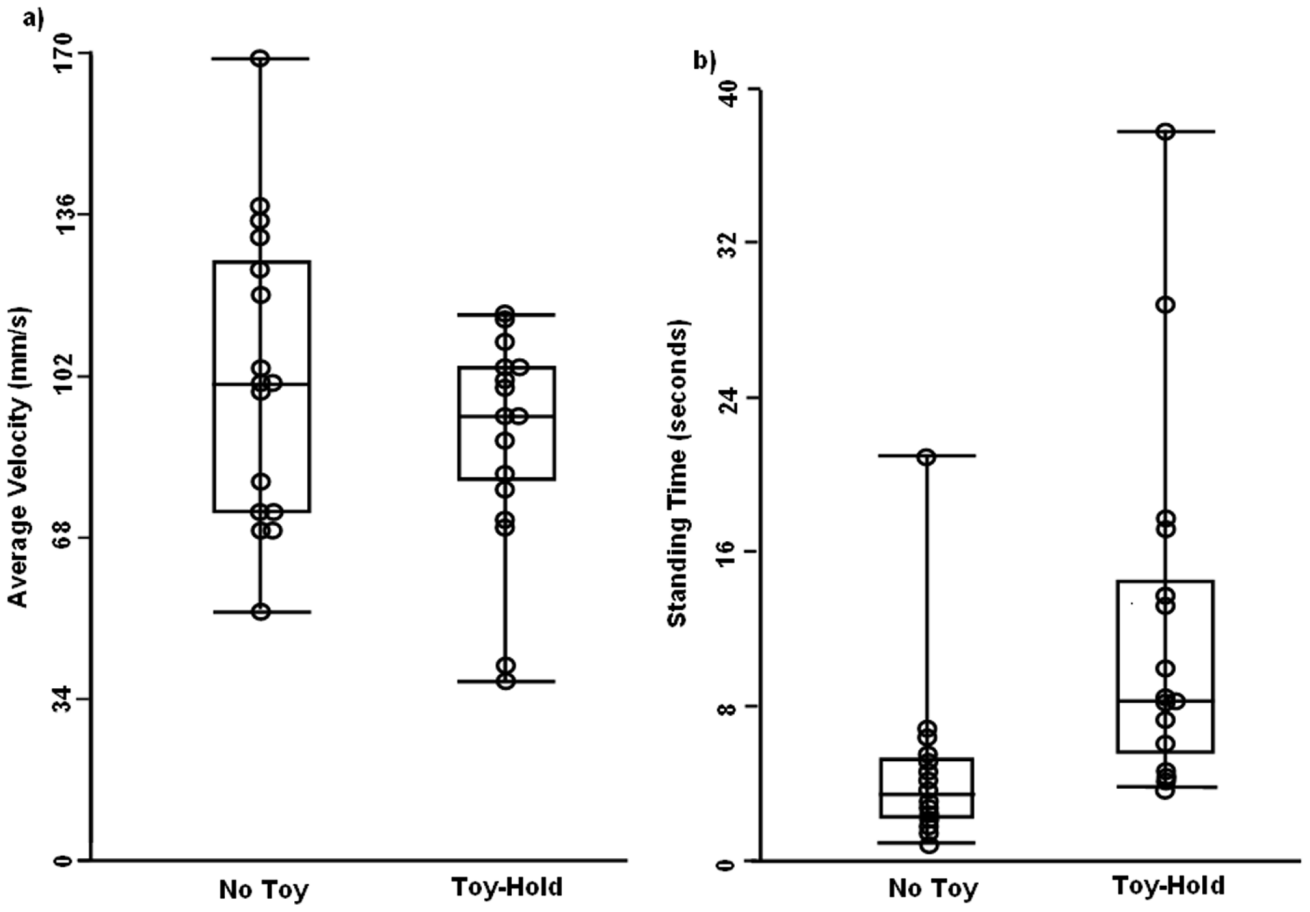
Box and whisker plot for the a) average center of pressure velocity and b) average standing time. The box encompasses the upper and lower quartile values of the data, and the whiskers show the range. The line in the center of the box is the median. The individual subject data is represented as circles superimposed on the plots. In the cases where individual subjects had near identical values, one circle is translated to the right for readability.

Infants stood on average, for 12.09 seconds (SE = 2.38) when holding a toy, but for only 4.71 seconds (SE = 1.17) when not holding a toy (Figure 2b); *t*(15) = 4.43, *p*<.001. Percent determinism (the outcome variable used from RQA) in the medial-lateral direction was 86.3% (SE = 1.58) in the no toy condition and 78.9% (SE = 3.37) in the toy-hold condition; *t*(15) = 2.324, *p*<.05. No significant difference in percent determinism was observed in the anterior-posterior direction between the toy (83.5%, SE = 2.37) and no-toy (85.9%, SE = 1.92) conditions; *t*(15) = .944, *p*>.05. CoPSD in the medial-lateral direction was 13.68 mm (SE = 1.02) in the no toy condition and 11.36 mm (SE = 1.02) in the toy-hold condition; *t*(15) = 2.938, *p*<.01. No significant difference in CoPSD was observed in the anterior-posterior direction between the toy (11.97 mm, SE = .768) and no-toy condition (12.08 mm, SE = .711); *t*(15) = .112, *p*>.05. The average CoP velocity was also lower when infants held a toy (89 mm/s, SE = 6) as compared to when not holding a toy (102 mm/s, SE = 8; Figure 2a); *t*(15) = 2.07, *p*<.05.

## Discussion

Our results suggest that despite limited standing experience, even newly standing stabilize standing posture when holding a toy. This stabilization was evident from the longer standing times, reduced CoP velocity, and lower magnitude of CoP movement in the ML direction. Interestingly, when holding a toy, percent determinism (as assessed using RQA), also decreased. However, when simply standing without holding the toy, infants exhibited their typical unstable body posture and stood for about one third the amount of time as in the toy-hold condition.

These results are congruent with Karasik, Adolph, Tamis-LeMonda, and Zuckerman [29]. As discussed previously, they found that infants fell twice as much when walking without carrying an object. The authors, surprised by this finding, stated, “An unexpected finding was that falls were more frequent while not carrying objects than while carrying.” Similar to our original assumptions, they assumed stability would decrease while carrying a toy. They posited two potential explanations for their surprising finding. First, they believed that the object in the hand may have focused the infant's attention. This focused attention ultimately improved walking stability. Second, the object may have been utilized by the infant as a crutch that ultimately improved stability. This possibility is consistent with the light-touch literature that has found lightly touching a surface, below the level that can be used for mechanical support, stabilizes posture, e.g. [35]. Both of these interpretations are appropriate here. Specifically, our infants were very attentive to the toys they were grasping. Additionally, grasping the objects did provide proprioceptive touch information.

To further examine this phenomenon of infant stabilization, future studies should attempt to decouple these two above explanations. For example, studies that motivate infants to stand while watching a computer monitor that displays attractive images would capture the infants attention without providing proprioceptive information. If infants did indeed become more stable while watching the computer monitor, these results would demonstrate infant stabilization occurs due to increased attention rather than light touch. Our study extends the findings of Karasik et al. [29] by demonstrating that grasping an object stabilizes posture before the onset of walking. This ability to stabilize posture when performing a goal-directed task is therefore acquired soon after the onset of independent stance and may ultimately be an important landmark in motor development in that it may provide infants with the ability to perform goal-directed locomotory tasks.

The time-dependent measures of sway provide insight into the mechanisms underlying the observed improvements in posture. Interestingly, the RQA and CoPSD measures revealed that postural sway in the medial-lateral direction was more sensitive than the anterior-posterior direction to task constraints. Previous adult literature has suggested that the mechanisms governing anterior-posterior sway is different than medial-lateral. Specifically, anterior-posterior sway is controlled by muscles about the ankle joints while medial-lateral sway is a loading/unloading mechanism controlled primarily by the hip musculature [36]. Similar to other dimensions of infant development such as reaching and growth [37], standing and the task-dependent postural control appears to develop along a proximal-distal trajectory. Where, the ability to stabilize posture to perform a goal-directed task develops first in the more proximal hip muscles and later in the ankle muscles.

In the current study, percent determinism (an outcome variable of the RQA analysis) was higher in the no-toy relative to the toy-hold condition in the medial-lateral direction. Less deterministic sway (as measured using RQA) has been interpreted in the adult literature to signify more healthy and adaptable postural dynamics [31], [32]. For example, the CoP of dancers has been found to be less deterministic than non-dancers [31] and the CoP of healthy individuals is less deterministic than individuals with Parkinson's disease [32]. These studies suggest that less deterministic sway patterns are characteristic of a healthy postural system during upright stance. Past research has also suggested that postural dynamics change within an individual based on the demands of a concurrent task [12]. This research suggests that in some circumstances, such as when performing a precision task, generating deterministic and predictable sway patterns helps maintain manual accuracy. However, less deterministic sway patterns, such as those observed in the current study, may be more indicative of healthy and adaptable postural sway dynamics. The less deterministic sway patterns may have allowed the infant to better attenuate any internally or externally generated perturbations to balance while holding the toy. These postural dynamics may contribute to the longer standing time observed in the toy-hold condition. This interpretation is further supported by the fact that there was a reduction in the total postural excursions in the medial-lateral direction (as measured by CoPSD) and a reduction in the velocity of sway. Thus, the shift in both the dynamics and magnitude of postural sway in the medial-lateral direction were conducive to holding the toy and suggests that task dependent postural control first develops about the hip musculature.

Adapting posture about the proximal degrees of freedom is different than what is typically observed in adults. Specifically, in the absence of a large perturbation, standing posture in adults is primarily regulated about the ankle joints [36]. Controlling posture about the hip would allow infants to shift body weight in a manner that allows them to stand longer, but would not afford them the level of control needed to exhibit adult like levels of task-dependent postural control. In future studies, it would be interesting to observe the age when the distal degrees of freedom contribute to task-dependent postural control and how control about the various postural degrees of freedom develops through childhood. For example, it would be interesting to examine if AP and ML sway when performing a goal-directed task while walking, e.g. [29] also emerges along a differing time course. Additionally, future studies should examine the kinematics of task-dependent postural control in young infants. Interesting multi-joint strategies that are not observable using traditional CoP analysis may be revealed through a kinematic analysis.

It is important to note that although infants stabilized balance; they were still relatively unstable compared to adults. Even when holding the toy, they only stood for 14-seconds. Interestingly, although these relatively sophisticated postural strategies emerge early after independent stance, the development of adult-like task-dependent postural control is relatively protracted, extending past the first decade of life [38]. Future longitudinal research should examine the origins of these strategies. For example, it is possible that the integration of posture and other goal-directed behaviors begins when independently sitting. However, a second possibility is this ability is rapidly learned after the onset of stance. The fact that infants altered sway in the medial lateral direction when holding a toy provides some evidence that some aspects of adaptive postural control are transferred between postures. For example, when sitting, degrees of freedom about the ankle joint are not used. Thus, infants must adapt posture primarily about the hip when interacting with objects in the environment. This may explain why the current findings show task-dependent posture is primarily controlled about the hip in newly standing infants.

This research has implications for the treatment of children with motor delays. For example, infants with Down syndrome often stand and walk much later than typically developing children, potentially impeding long term motor function. Therefore, therapy and exercises are often performed so that children with Down syndrome develop the ability to independently walk within a more typical time frame [39]. These exercises are not always easy to conduct since children with Down syndrome have difficulty attending to directions [40]. However, cuing children to stand longer by manipulating a toy could provide an easy way to strengthen the child's leg and trunk muscles so that independent stance and walking manifest within a more typical time frame. Prompting children with motor delays to adopt a more stable stance may also help improve their confidence by allowing them to realize that they are capable of generating the necessary body stability to perform more mature motor behaviors such as walking. Anecdotally, one of the children in this study took her first independent steps immediately after attending to the toy. The reduction in body sway that was attained by attending to the toy may have facilitated stability, allowing the infant to attempt a new behavior.

In conclusion, when given a toy to hold, infants stabilized themselves showing that they possess better balance abilities than what has traditionally been believed. Neuromuscular limitations therefore cannot fully explain the unstable balance typically observed in infants. Rather, the goal of the infants should be considered when assessing or examining bipedal development.
